# Brevibacillin 2V, a Novel Antimicrobial Lipopeptide With an Exceptionally Low Hemolytic Activity

**DOI:** 10.3389/fmicb.2021.693725

**Published:** 2021-06-17

**Authors:** Xinghong Zhao, Xiaoqi Wang, Rhythm Shukla, Raj Kumar, Markus Weingarth, Eefjan Breukink, Oscar P. Kuipers

**Affiliations:** ^1^Department of Molecular Genetics, Groningen Biomolecular Sciences and Biotechnology Institute, University of Groningen, Groningen, Netherlands; ^2^Membrane Biochemistry and Biophysics, Bijvoet Centre for Biomolecular Research, Department of Chemistry, Faculty of Science, Utrecht University, Utrecht, Netherlands; ^3^NMR Spectroscopy, Bijvoet Centre for Biomolecular Research, Department of Chemistry, Faculty of Science, Utrecht University, Utrecht, Netherlands

**Keywords:** antimicrobial activity, lipopeptide, NRPS, brevibacillin, *Brevibacillus*, *Enterococcus*, *Staphylococcus*

## Abstract

Bacterial non-ribosomally produced peptides (NRPs) form a rich source of antibiotics, including more than 20 of these antibiotics that are used in the clinic, such as penicillin G, colistin, vancomycin, and chloramphenicol. Here we report the identification, purification, and characterization of a novel NRP, i.e., brevibacillin 2V (lipo-tridecapeptide), from *Brevibacillus laterosporus* DSM 25. Brevibacillin 2V has a strong antimicrobial activity against Gram-positive bacterial pathogens (minimum inhibitory concentration = 2 mg/L), including difficult-to-treat antibiotic-resistant *Enterococcus faecium*, *Enterococcus faecalis*, and *Staphylococcus aureus*. Notably, brevibacillin 2V has a much lower hemolytic activity (HC_50_ > 128 mg/L) and cytotoxicity (CC_50_ = 45.49 ± 0.24 mg/L) to eukaryotic cells than previously reported NRPs of the lipo-tridecapeptide family, including other brevibacillins, which makes it a promising candidate for antibiotic development. In addition, our results demonstrate that brevibacillins display a synergistic action with established antibiotics against Gram-negative bacterial pathogens. Probably due to the presence of non-canonical amino acids and D-amino acids, brevibacillin 2V showed good stability in human plasma. Thus, we identified and characterized a novel and promising antimicrobial candidate (brevibacillin 2V) with low hemolytic activity and cytotoxicity, which can be used either on its own or as a template for further total synthesis and modification.

## Introduction

The non-ribosomally produced peptides (NRPs) have formed a rich source of antimicrobials, including more than 20 marketed antibiotics, such as chloramphenicol, penicillin G, colistin, and vancomycin (Süssmuth and Mainz, 2017). Among these, lipopeptides form a rich class of NRPs that have shown a strong antimicrobial activity against Gram-positive (Hamamoto et al., 2015; Ling et al., 2015) and/or Gram-negative (Cochrane et al., 2014; Cociancich et al., 2015) pathogens.

Large numbers of antimicrobial NRPs have been isolated from different *Brevibacillus* spp. (Yang and Yousef, 2018), including many linear lipopeptides, such as bogorol A–E (Barsby et al., 2001, 2006), brevibacillin (Yang et al., 2016), and brevibacillin V (Wu et al., 2019). Bogorol A–E, brevibacillin, and brevibacillin V are produced by *Brevibacillus laterosporus* PNG-276, *B. laterosporus* OSY-I1, and *B. laterosporus* fmb70 (Barsby et al., 2001, 2006; Yang et al., 2016; Wu et al., 2019), respectively, and all of them can be grouped into the family of non-ribosomally produced linear lipo-tridecapeptides (Yang and Yousef, 2018). These lipo-tridecapeptides show a strong antimicrobial activity against Gram-positive pathogenic bacteria, including methicillin-resistant *Staphylococcus aureus* and vancomycin-resistant *Enterococcus* spp. (Barsby et al., 2006; Yang et al., 2016; Wu et al., 2019). However, many lipo-tridecapeptides have the disadvantage of being highly hemolytic and cytotoxic (Li et al., 2018), which have limited the potential of many antimicrobial peptides to be developed as therapeutics (Mor and Nicolas, 1994; Ciornei et al., 2005; Andraö et al., 2007; Savoia et al., 2008). Due to the presence of non-canonical amino acids, D-amino acids, and several other modifications, NRPs have commonly shown good proteolytic stability. Therefore, it is of interest to discover and further develop lipo-tridecapeptides with low hemolytic activity and cytotoxicity as potential antimicrobial agents.

In this study, four lipo-tridecapeptides were isolated from *B. laterosporus* DSM 25. Two of these compounds are known peptides, i.e., brevibacillin and brevibacillin V, while the other two compounds are novel lipo-tridecapeptides that we named brevibacillin I and brevibacillin 2V. All these four lipo-tridecapeptides showed a strong antimicrobial activity against Gram-positive pathogenic bacteria and showed synergistic effects with marketed antibiotics against Gram-negative pathogens. In addition, brevibacillin 2V showed good stability in human plasma. Notably, brevibacillin 2V did not exhibit a hemolytic activity when present at 128 mg/L, demonstrating its potency to be further studied and developed as a candidate antimicrobial agent for controlling specific antibiotic-resistant bacterial pathogens.

## Materials and Methods

### Bacterial Strains Used and Growth Conditions

The strains used in this study are listed in Supplementary Table 1. *B. laterosporus* DSM 25 cells were inoculated in LB and incubated at 37°C to prepare an overnight culture. For the production of brevibacillins, an overnight culture of *B. laterosporus* DSM 25 cells was inoculated (50-fold dilution) in minimal expression medium (MEM) and grown at 30°C for 36 h. All indicator strains were inoculated in LB and incubated at 37°C to prepare overnight cultures.

### Purification of Cationic Peptides From a *B. laterosporus* DSM25 Culture

An overnight culture of *B. laterosporus* DSM 25 cells was inoculated in MEM and grown at 30°C for 36 h. Subsequently, the culture was centrifuged at 15,000 *g* for 15 min; the supernatant was collected and the pH was adjusted to 7.2 with a saturated sodium hydroxide solution. After that, the culture was applied to a CM Sephadex^TM^ C-25 column (GE Healthcare) equilibrated with distilled water. The flow-through was discarded, and the column was subsequently washed with 12 column volumes (CV) of distilled water. The peptide was eluted with 6 CV 2 M NaCl. The eluted peptide was then applied to a SIGMA-ALDRICH C18 Silica gel spherically equilibrated with 10 CV of Milli-Q water containing 5% (v/v) MeCN and 0.1% (v/v) trifluoroacetic acid. After washing with 10 CV of Milli-Q water containing 5% MeCN and 0.1% trifluoroacetic acid, the peptide was eluted from the column using up to 10 CV of Milli-Q water containing 50% MeCN and 0.1% trifluoroacetic acid. Fractions containing the eluted peptide were freeze-dried and dissolved in Milli-Q water. After filtration through a 0.2-μm filter, the cationic peptides were purified on an Agilent 1260 Infinity high-performance liquid chromatography (HPLC) system with a Phenomenex Aeris^TM^ C18 column (250 × 4.6 mm, 3.6 μm particle size, 100 Å pore size). Acetonitrile was used as the mobile phase, and a gradient of 30–45% MeCN/Milli-Q water (v/v; containing 0.1% trifluoroacetic acid) over 30 min at 1 ml per min was used for separation. The purified lipo-tridecapeptides were eluted with between 35 and 40% MeCN/Milli-Q water (v/v; containing 0.1% trifluoroacetic acid). The yields for brevibacillin, brevibacillin V, brevibacillin 2V, and brevibacillin I were 5, 3, 1.5, and 0.5 mg/L, respectively.

### Mass Spectrometry

Matrix-assisted laser desorption ionization-time-of-flight (MALDI-TOF) mass spectrometer analysis was performed using a 4800 Plus MALDI TOF/TOF Analyzer (Applied Biosystems) in the linear-positive mode as in previous studies (Zhao et al., 2020a,b,c). Briefly, a 1-μl sample was spotted on the target and dried at room temperature. Subsequently, 0.6 μl of matrix solution (5 mg/ml of α-cyano-4-hydroxycinnamic acid) was spotted on each sample. After the samples had dried, MALDI-TOF MS was performed.

### Liquid Chromatography–Tandem Mass Spectrometry Analysis

Since liquid chromatography–tandem mass spectrometry (LC–MS/MS) can be used to yield b and y ions for peptides, it is widely used in peptide structure elucidation (McClerren et al., 2006; Zhang et al., 2014; Zhao et al., 2020b). To gain insight into the molecular structures of the peptides, LC–MS/MS was performed using a Q-Exactive mass spectrometer fitted with an Ultimate 3000 UPLC, an ACQUITY BEH C18 column (2.1 × 50 mm, 1.7 μm particle size, 200 Å; Waters), a HESI ion source, and a Orbitrap detector. A gradient of 5–90% MeCN/Milli-Q water (v/v) with 0.1% formic acid (v/v), at a flow rate of 0.35 ml/min over 60 min, was used. MS/MS was performed in a separate run in parallel reaction monitoring mode, selecting the doubly and triply charged ion of the compound of interest.

### Spot-on-Lawn Assay

Overnight-cultured *S. aureus* ATCC15975 (MRSA) was added to 0.7% Mueller–Hinton agar (w/v, temperature 42°C) at a final concentration of 0.25% (v/v), and then the mixture was poured onto plates, with 10 ml for each 8.5-cm-diameter circular plate or 20 ml for each 9.5-cm square plate. Subsequently, a spot-on-lawn assay was used to analyze the antimicrobial activity (Zhao et al., 2020c). In short, antibiotics were loaded to the agar plate that contains an indicator strain. After the antibiotic solution drops had dried, the plates were transferred to a 37°C incubator for overnight incubation.

### Minimum Inhibitory Concentration Assay

Minimum inhibitory concentration (MIC) values were determined by using broth micro-dilution according to the standard guidelines (Wiegand et al., 2008). Briefly, the test medium was cation-adjusted Mueller–Hinton broth. Cell concentration was adjusted to approximately 5 × 10^5^ cells per milliliter. After 20 h of incubation at 37°C, the MIC was defined as the lowest concentration of antibiotic with no visible growth (Zhao et al., 2020c). Each experiment was performed in triplicate.

### Hemolytic Activity

This assay was performed as per the method described in previous studies (Ling et al., 2015; Li et al., 2018). Briefly, erythrocytes were isolated from a healthy human volunteer donor (ordered from Sanquin, Netherlands^1^) and washed with phosphate-buffered saline (PBS) three times. After that, peptides were added at final concentrations of 128, 64, 32, 16, 8, 4, 2, 1, and 0 mg/L in PBS containing 2% (v/v) erythrocytes. The cells were incubated at 37°C for 1 h and centrifuged for 5 min at 8,000 *g*. The supernatant was transferred to a 96-well plate, and the absorbance was measured at a wavelength of 450 nm with a Thermo Scientific Varioskan^TM^ LUX multimode microplate reader. The absorbance relative to the positive control, which was treated with 10% Triton X-100, was defined as the percentage of hemolysis. The 50% human blood cell hemolysis concentration (HC_50_) of peptides was calculated as described in previous studies (Reed and Muench, 1938).

### Mammalian Cytotoxicity

The cytotoxicity of brevibacillins was evaluated on HepG2 cells by using the XTT (Cell Proliferation Kit XTT, AppliChem) assay (Roehm et al., 1991). HepG2 cells [in Dulbecco’s modified Eagle’s medium (DMEM) supplemented with 10% fetal bovine serum] were seeded into 96-well plates and incubated at 37°C with 5% CO_2_. After 24 h, the medium was replaced with a fresh medium (DMEM with 2% FBS, 100 μl per well) containing different concentrations of brevibacillins (Zhao et al., 2017). After 24 h of incubation, the XTT reagent was added to the cultures according to the manufacturer’s instructions, and the plates were incubated at 37°C for 2 h with 5% CO_2_. Subsequently, the absorbance values were measured by using a Varioskan^TM^ LUX multimode microplate reader (Thermo Fisher Scientific) at 485 nm (reference, 690 nm). The 50% cell toxicity (CC_50_) of peptides was calculated as described in a previous study (Reed and Muench, 1938).

### Synergy Assay

The synergistic effect of brevibacillins with established antibiotics was monitored using the checkerboard method (Doern, 2014). Briefly, antibiotics were diluted in twofold series, while brevibacillins were added at a certain final concentration of 1, 2, or 4 mg/L. Indicator strains were added at a final concentration of 5 × 10^5^ cfu/ml. After incubation at 37°C for 20 h, the OD_600_ of plates was determined. The fractional inhibitory concentration index (FICI) was calculated using the following formula: FICI = (MIC compound A in combination with B / MIC compound A) + (MIC compound B in combination with A / MIC compound B). The FICI value suggests a synergistic (≤0.5), addictive (>0.5–1), no interaction (1–4), and antagonism (>4) effect of the two compounds (Doern, 2014).

### Plasma Stability Assay

For plasma stability tests, brevibacillin and brevibacillin 2V were lyophilized and resuspended in plasma (isolated from a healthy human volunteer donor; ordered from Sanquin, Netherlands; see text footnote 1) at a final concentration of 200 mg/L. After that, the peptides were incubated with shaking (200 rpm) for 8 h (this evaluation time was set based on a continuing study, which showed that brevibacillins killed all *S. aureus* cells in 4 h) at 37°C. The samples were collected at desired time points (0, 1, 2, 4, and 8 h) and stored at −80°C. After the collection of samples of the last time point, the antimicrobial activity of all samples against *S. aureus* (MRSA) was investigated by a spot-on-lawn assay as per the method described above. The reduction in the size of the halo was related to the degradation of the peptide (Li et al., 2021).

### Quantification and Statistical Analyses

GraphPad Prism 7.0 was used to fit the data of hemolytic assay and cytotoxicity assay in Figure 3. The experiments were conducted in triplicate, and data are presented as the mean value of triplicate experiments. Data Explorer Software was used to analyze the MALDI-TOF data; Thermo Scientific Xcalibur software was used to analyze the LC–MS/MS data. The statistical significance of the data was assessed using Duncan’s multiple-range test with the software SPSS Statistics 19.0. *P*-values less than 0.05 were considered to be statistically significant.

**FIGURE 1:**
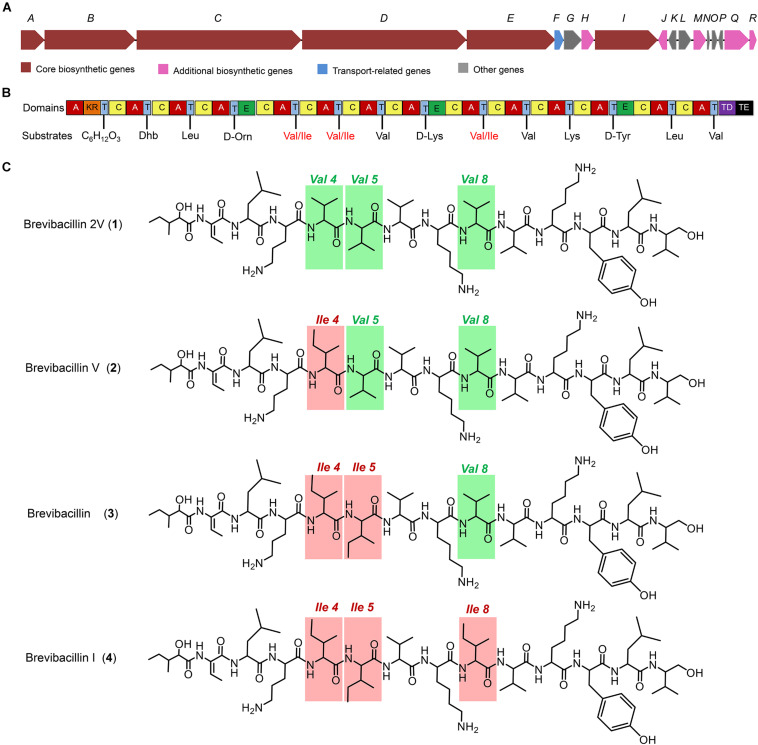
The structures of brevibacillins and the predicted biosynthetic gene cluster. **(A)** The non-ribosomal peptide synthetase genes harbored by the *Brevibacillus laterosporus* DSM 25 genome. **(B)** The catalytic domains encoded by the gene cluster and the substrates incorporated by the respective modules. Domains: A, adenylation; KR, ketoreductase; T, thiolation; C, condensation; E, epimerization; TD, terminal reductase; TE, thioesterase. **(C)** Structures of brevibacillins. The red rectangles indicate the more hydrophobic amino acid residues of brevibacillin V, brevibacillin, and brevibacillin I relative to brevibacillin 2V.

**FIGURE 2:**
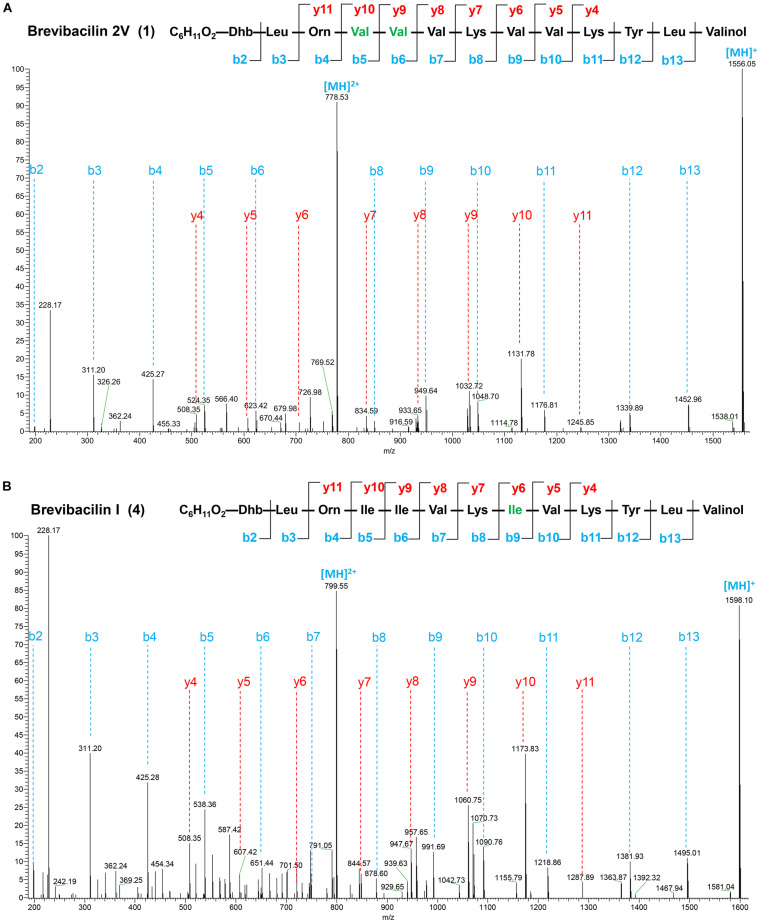
Liquid chromatography-tandem mass spectrometry (LC–MS/MS) spectra and the proposed structures of brevibacillin 2V **(A)** and brevibacillin I **(B)**. Fragment ions are indicated. LC–MS/MS analysis was performed using a Q-Exactive mass spectrometer fitted with an Ultimate 3000 UPLC, an ACQUITY BEH C18 column (2.1 × 50 mm, 1.7 μm particle size, 200 Å; Waters), a HESI ion source, and a Orbitrap detector. MS/MS was performed in a separate run in parallel reaction monitoring mode, selecting the doubly and triply charged ion of the peptides. The structures of brevibacillin 2V and brevibacillin I were elucidated by using known lipo-tridecapeptides as reference templates.

**FIGURE 3:**
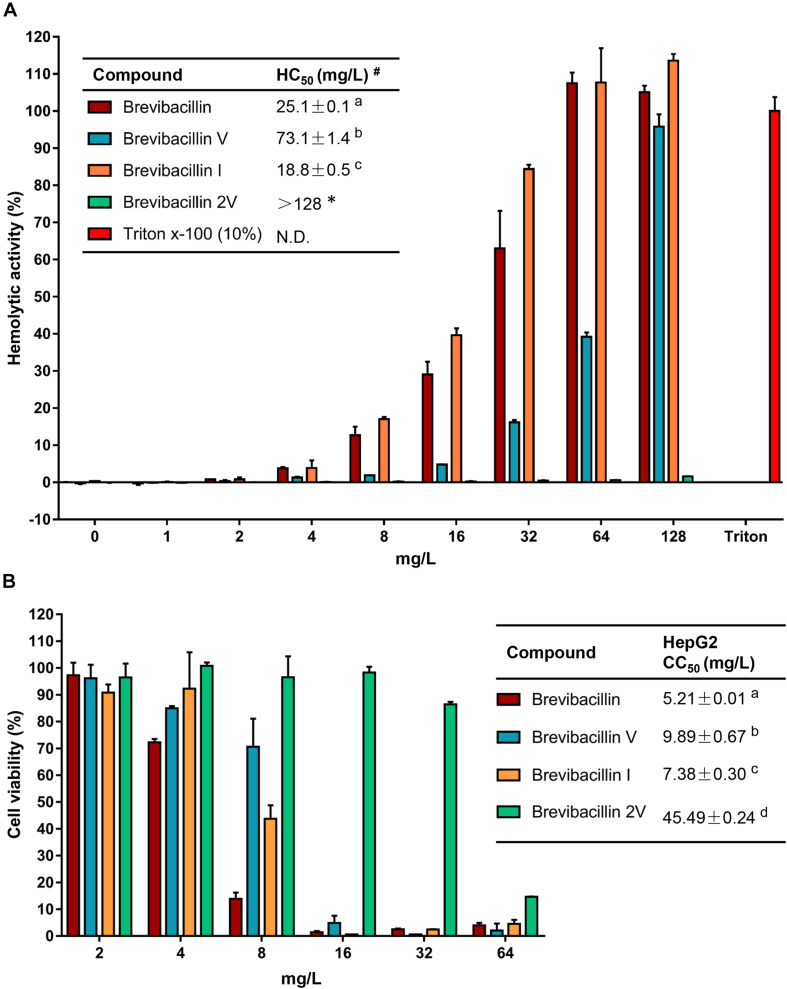
Brevibacillins activities against eukaryotic cells. **(A)** Human erythrocytes were incubated with brevibacillins at concentrations ranging from 1 to 128 mg/L. Their hemolytic activity was assessed by the release of hemoglobin. Cells treated without a tested compound were used as no lysis control. Cells treated with 10% Triton X-100 were used as complete lysis control. The data are representative of three independent experiments. ^#^HC_50_ is the concentration that causes 50% hemolysis of human red blood cells. N.D., not determined. ^*a,b,c*^Significant differences exist when not the same letters are indicated in the HC_50_ column (*p* < 0.05). *No hemolytic activity was observed for brevibacillin 2V at 128 mg/L and its HC_50_> 128 mg/L. **(B)** Cytotoxicity of brevibacillins against HepG2 cells. HepG2 cells were treated with brevibacillins at concentrations ranging from 2 to 64 mg/L. Cells treated without a tested compound were used as untreated control. After 24 h of treatment, cell viability was determined by an XTT kit. The data are representative of three independent experiments. ^a,b,c,d^Significant differences exist when not the same letters are indicated in the CC_50_ column (*p* < 0.05).

## Results

### Characterization of Peptides Discovered by Genome Mining

Many lipopeptide antibiotics that originate from *Brevibacillus* spp. (Barsby et al., 2001, 2006; Yang et al., 2016; Yang and Yousef, 2018; Wu et al., 2019) have been discovered. In a previous study, a cyclic lipopeptide, brevicidine, was discovered from *B. laterosporus* DSM 25 by genome mining, which showed an antimicrobial activity against Gram-negative pathogenic bacteria (Li et al., 2018; Zhao et al., 2020b). More recently, brevicidineB, a natural analog of brevicidine, was discovered from *B. laterosporus* DSM 25, which showed an extended target specificity, showing an antimicrobial activity against both Gram-negative and Gram-positive pathogens (Zhao and Kuipers, 2021). In the present study, we found, by genome mining with the assistance of antiSMASH (Medema et al., 2011; Blin et al., 2019), that the genome (NCBI GenBank accession: GCA_002706795.1) of *B. laterosporus* DSM 25 contains a gene cluster (NCBI GenBank accession: KY810814.1.) for the synthesis of lipo-tridecapeptides (Figures 1A,B). *B. laterosporus* DSM 25 produced several cationic peptides that were purified by a CM Sephadex^TM^ C-25 column (GE Healthcare). After HPLC purification (Supplementary Figure 1), MALDI-TOF MS was used to measure the molecular weight of the purified peptides. Four compounds with a mass between 1,550 and 1,600 Da were found, i.e., compound 1, 1,555 Da; compound 2, 1,569 Da; compound 3, 1,583 Da; and compound 4, 1,597 Da (Supplementary Figures 1, 2), which potentially belong to the lipo-tridecapeptide family. Further characterization of these peptides was performed by LC–MS/MS analysis, and the results are shown in Figure 2 and Supplementary Figures 3, 4. The structures of the four compounds were elucidated by using known lipo-tridecapeptides as reference templates (Barsby et al., 2001, 2006; Yang et al., 2016; Yang and Yousef, 2018; Wu et al., 2019; Li et al., 2020). Two of these compounds are known peptides, i.e., brevibacillin (compound 3) and brevibacillin V (compound 2) (Yang et al., 2016; Wu et al., 2019), while the other two compounds are novel lipo-tridecapeptides that we named brevibacillin I (compound 4) and brevibacillin 2V (compound 1) (Figure 1C). In previous studies, brevibacillin and brevibacillin V showed a strong antimicrobial activity against Gram-positive bacteria (Yang et al., 2016; Wu et al., 2019). To investigate if the newly discovered brevibacillin I and brevibacillin 2V have similar or different antimicrobial activity with the known brevibacillins, we performed a spot-on-lawn assay using *S. aureus* ATCC15975 (MRSA) as an indicator strain. Nisin was used as a positive control. The results show that all brevibacillins have a comparable antimicrobial activity to each other against *S. aureus* (MRSA). In addition, they have a stronger antimicrobial activity against *S. aureus* (MRSA) than the well-known lantibiotic nisin (Supplementary Figure 5).

### Brevibacillins Show a Strong Antimicrobial Activity Against Gram-Positive Bacterial Pathogens

After the spot-on-lawn assays have shown that brevibacillins have a good antimicrobial activity against *S. aureus* (MRSA), the antimicrobial activity of these lipo-tridecapeptides against pathogenic bacteria was further evaluated by MIC assays. All of the brevibacillins showed a strong antimicrobial activity against tested Gram-positive pathogenic bacteria, with a MIC value of 1–2 mg/L, including difficult-to-treat antibiotic-resistant *Enterococcus faecium*, *Enterococcus faecalis*, and *S. aureus* (Table 1). The similar antimicrobial activities indicate that the varying amino acid residues in the different lipo-tridecapeptides have no significant influence on their antimicrobial activity. In addition, brevibacillins showed an antimicrobial activity against Gram-negative pathogenic bacteria, yet these activities were much lower compared to the antimicrobial activity against Gram-positive bacteria (Table 1). These results are consistent with previous studies which show that lipo-tridecapeptides have an antimicrobial activity against Gram-positive bacteria but have an insufficient antimicrobial activity against Gram-negative bacteria (Barsby et al., 2001, 2006; Yang et al., 2016; Wu et al., 2019).

**TABLE 1:** Minimum inhibitory concentration (MIC) values of brevibacillins against pathogenic bacteria.

**Microorganism**	**MIC (mg/L)^a^**					
	**Bre**	**Bre V**	**Bre I**	**Bre 2V**	**Poly B**	**Nisin**
**Gram-positive pathogenic bacteria**						
*Staphylococcus aureus* ATCC15975 (MRSA)	2	1–2	2	2	32	4–8
*Enterococcus faecium* LMG16003 (VRE)	2	2	2	2	16	4
*Enterococcus faecalis* LMG16216 (VRE)	2	2	2	2	16	4
*Bacillus cereus* ATCC14579	1	1	2	2	8	4
**Gram-negative pathogenic bacteria**						
*Acinetobacter baumannii* ATCC17978	32	64	64	32	4	32
*Escherichia coli* ATCC25922	32	32	32	16	2	64
*Pseudomonas aeruginosa* LMG6395	64	64	64	64	1	128
*Klebsiella pneumoniae* LMG20218	32	64	64	32	2	128

*^*a*^The MIC was determined by broth microdilution.*

*VRE, vancomycin-resistant *Enterococcus*; MRSA, methicillin-resistant *S. aureus*; Bre, brevibacillin; Bre V, brevibacillin V; Bre I, brevibacillin I; Bre 2V, brevibacillin 2V; Poly B, polymyxin B.*

### Brevibacillin 2V Does Not Exhibit Hemolytic Activity When Present at 128 mg/L

To assess in an initial test the safety of brevibacillins to human beings or animals, the hemolytic activity of brevibacillins to human blood cells and the cytotoxicity of brevibacillins to a human liver cell line (HepG2) were monitored. For the hemolytic activity assay, human blood cells were incubated in the presence of brevibacillin concentrations ranging from 1 to 128 mg/L. After incubation at 37°C for 1 h, the OD_450_ of the supernatants was measured, and the hemolytic activities of the brevibacillins were calculated as described in previous studies (Ling et al., 2015; Li et al., 2018). Brevibacillin, brevibacillin V, and brevibacillin I showed a significant hemolytic activity at concentrations that are two- to fourfold higher than their MIC values (Figure 3A and Table 1), with a half red blood cell hemolysis (HC_50_) concentration of 18.8 ± 0.5, 73.1 ± 1.4, and 25.1 ± 3.9, respectively. In addition, in the presence of either 64 mg/L of brevibacillin/brevibacillin I or 128 mg/L of brevibacillin V, human blood cells were completely lysed (Figure 3A). In contrast, brevibacillin 2V showed no hemolytic activity against human red blood cells at a high concentration of 128 mg/L (Figure 3A).

For cytotoxicity assays, HepG2 cells were incubated in the presence of brevibacillin concentrations ranging from 2 to 64 mg/L. After 24 h of incubation, cell viability was determined using an XTT kit. Under the experimental conditions used, brevibacillin, brevibacillin V, and brevibacillin I showed a high cytotoxicity to HepG2 cells with a half cell toxicity (CC_50_) value of 5–10 mg/L (Figure 3B), which is only 2.5- to fivefold of their MIC values against the bacterial pathogens (Figure 3B and Table 1). However, brevibacillin 2V showed much lower cytotoxicity than the other brevibacillins with a CC_50_ value of 45.5 mg/L against HepG2 cells, which is 23-fold higher than its MIC values against bacterial pathogens (Figure 3B and Table 1). In the presence of brevibacillin, brevibacillin V, or brevibacillin I at a concentration of 16 mg/L, all of the HepG2 cells were killed. These results are consistent with a previous study which shows that bogorol has high cytotoxicity with a CC_50_ of below 8 mg/L (Li et al., 2018). Together these results, with their high hemolytic activity, indicate that brevibacillin, brevibacillin V, brevibacillin I, and bogorol are too toxic to be developed as antibiotics for *in vivo* therapy. In contrast, brevibacillin 2V showed no cytotoxicity at the relatively high concentration of 32 mg/L (Figure 3B). Together with its non-hemolytic activity and low cytotoxicity, brevibacillin 2V shows a high potential, justifying its further study and development as an alternative antibiotic for controlling antibiotic-resistant bacterial pathogens.

### Brevibacillins Show a Synergistic Activity With Other Antibiotics Against Gram-Negative Pathogenic Bacteria

Brevibacillins show a strong antimicrobial activity against Gram-positive bacterial pathogens but have insufficient antimicrobial activity against Gram-negative bacterial pathogens (Table 1). To expand the antimicrobial potential of brevibacillins, we investigated the synergistic activity of brevibacillins with some antibiotics (nalidixic acid, azithromycin, rifampicin, and amikacin) against Gram-negative pathogens. The synergistic effect of peptides was evaluated by determining a FICI value, which can indicate either a synergistic (≤0.5), addictive (>0.5–1), no interaction (1–4), or antagonistic (>4) effect of the two compounds (Doern, 2014). The results are shown in Table 2 and Supplementary Tables 2–4. Brevibacillin, brevibacillin V, brevibacillin I, and brevibacillin 2V showed a synergistic activity with the tested antibiotics against corresponding Gram-negative pathogens. Most importantly, brevibacillins showed the highest synergistic activity with amikacin against *Acinetobacter baumannii* in all tests (Table 2 and Supplementary Tables 2–4), with *A. baumannii* being one of the three critical priority pathogens for R&D of new antibiotics (World Health Organization, 2017). The MIC value of amikacin against *A. baumannii* decreased by 32–64 folds in the presence of 4 mg/L brevibacillins (Table 2 and Supplementary Tables 2–4). Together with its non-hemolytic activity, low cytotoxicity, and the effective low MIC values, brevibacillin 2V shows a high potential to be developed as an adjuvant for other antibiotics to treat Gram-negative bacterial pathogen infections.

**TABLE 2:** Synergistic effect between brevibacillin 2V and antibiotics.

**Microorganism**	**Antibiotic**	**Minimum inhibitory concentration (MIC; mg/L) at various brevibacillin 2V concentrations^a^**				**FICI**
		**0**	**1**	**2**	**4**	
*E. coli* ATCC 25922	Nalidixic acid	2	0.5	0.5	0.125	0.313
	Rifampicin	4	2	2	0.25	0.313
	Amikacin	4	0.5	0.5	0.25	0.188
	Azithromycin	2	1	0.5	0.06	0.281
*A. baumannii* ATCC17978	Nalidixic acid	32	16	16	16	0.531
	Rifampicin	32	16	16	8	0.375
	Amikacin	16	8	4	*0.25*	*0.141*
	Azithromycin	16	8	8	8	0.531
*P. aeruginosa* LMG6395	Nalidixic acid	256	64	64	64	0.266
	Rifampicin	32	16	16	16	0.516
	Amikacin	2	0.5	0.5	0.5	0.266
	Azithromycin	128	32	32	32	0.266
*K. pneumoniae* LMG20218	Nalidixic acid	32	16	16	16	0.531
	Rifampicin	64	16	16	8	0.250
	Amikacin	0.5	0.5	0.25	0.25	0.563
	Azithromycin	16	16	16	16	1.031

*^*a*^The MIC was determined by broth microdilution.*

*FICI, fractional inhibitory concentration index [FICI = (MIC compound A in combination with B / MIC compound A) + (MIC compound B in combination with A / MIC compound B)].*

*The FICI value suggests a synergistic (≤0.5), addictive (>0.5–1), no interaction (1–4), and antagonism (>4) effect of the two compounds. The italic font indicates the best synergy effect among the tests.*

### Brevibacillins Show Good Stability in Plasma

**FIGURE 4:**
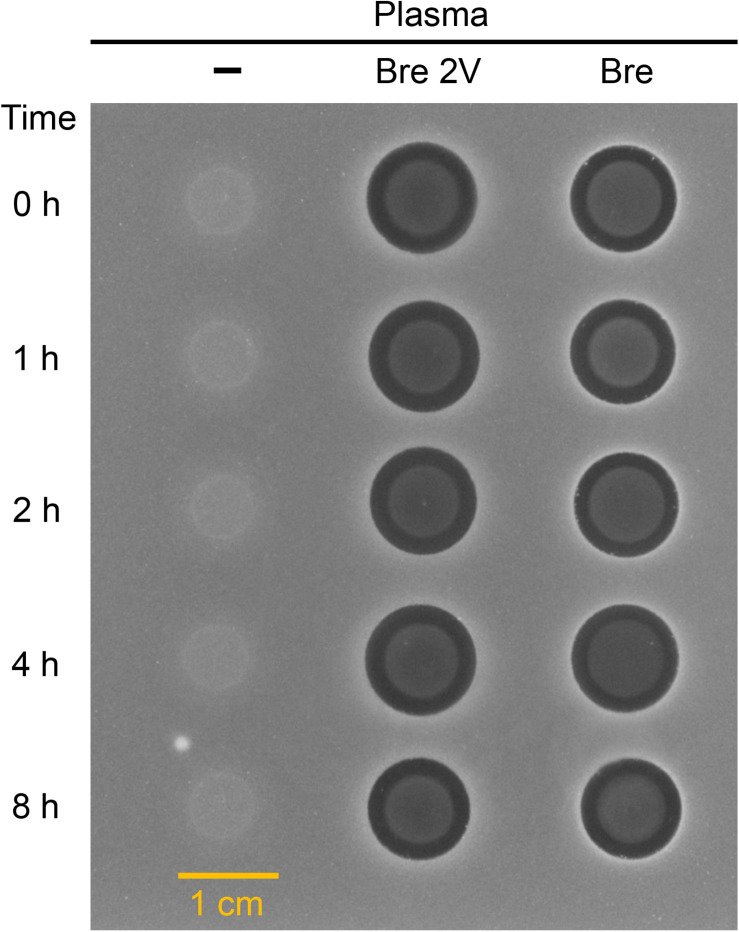
Antimicrobial activity of brevibacillin 2V brevibacillin against *Staphylococcus aureus* (MRSA) after exposure to human plasma. The starting concentration was 200 mg/L for both brevibacillin 2V and brevibacillin. Samples were collected at 0, 1, 2, 4, and 8 h, and 6 μl of each sample was loaded to a square plate containing *S. aureus* (MRSA). Bre 2V, brevibacillin 2V; Bre, brevibacillin.

In view of the therapeutic development potential of brevibacillin 2V, the stability of brevibacillin 2V and brevibacillin (highest yield among brevibacillins; to be used as a control to compare with brevibacillin 2V) in human plasma was investigated, using the method described in a previous study (Li et al., 2021). Brevibacillins were incubated in human plasma, and the reduction in antimicrobial activity was measured by spot-on-lawn assays against *S. aureus* (MRSA). Brevibacillins showed good stability in plasma during 4 h after exposure since no reduction in the sizes of the halos was observed (Figure 4). After 8 h of exposure in plasma, the sizes of the halos for brevibacillin 2V and brevibacillin only decreased a little bit (halo diameter decreased from 11 to 10 mm for brevibacillin 2V and from 11 to 10.5 mm for brevibacillin) (Figure 4). The good plasma stability of brevibacillin 2V might be due to the presence of non-canonical amino acids and D-amino acids, which are highly resistant to proteases (Li et al., 2021). These results demonstrate that brevibacillin 2V has good stability in plasma, which makes it more attractive for development as an antibiotic candidate.

## Discussion

**FIGURE 5:**
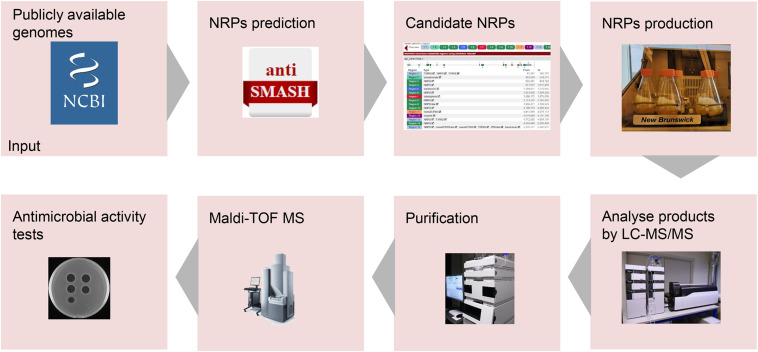
Pipeline for the development of novel non-ribosomally produced peptide (NRP)-based antimicrobials. Following the arrows, the process starts with the identification of NRP clusters. Next, the predicted NRPs have to be produced, isolated, and characterized. Finally, the antimicrobial activity of the purified NRPs will be determined.

Genome mining is a practical approach for discovering bioactive natural products from microorganisms (Montalbán-López et al., 2021). Genome mining tools with different specific uses have been developed, including BAGEL4 (van Heel et al., 2018), antiSMASH (Blin et al., 2019), and RiPP-PRISM (Skinnider et al., 2015). AntiSMASH is a well-known genome mining tool for discovering NRPs with antimicrobial activity (Blin et al., 2019). For instance, two non-ribosomally produced cyclic lipopeptides, i.e., brevicidine and laterocidine, were discovered by genome mining with the assistance of antiSMASH (Li et al., 2018). In this study, a lipo-tridecapeptide synthetic gene cluster was discovered from *B. laterosporus* DSM 25 by genome mining with the assistance of antiSMASH (Medema et al., 2011; Blin et al., 2019). Subsequently, antimicrobial lipo-tridecapeptides were isolated and identified by following the pipeline in Figure 5.

In this study, we isolated the predicted lipo-tridecapeptides from the supernatant of *B. laterosporus* DSM 25 culture. This allows an iron change-based CM Sephadex^TM^ C-25 column to be applied to purify the produced products since lipo-tridecapeptides are cationic peptides. In contrast, lipo-tridecapeptides were previously isolated from bacterial cells (Barsby et al., 2001, 2006; Yang et al., 2016), which is more difficult to process. Thus, the here found lipo-tridecapeptides are much more readily purified than the previously reported lipo-tridecapeptides. For elucidation of the purified lipo-tridecapeptides structures, we used the previously reported lipo-tridecapeptides as templates. Such template-based structure elucidation approach has been successfully used in previous studies to elucidate peptide structures (Barsby et al., 2006; Wu et al., 2019). These results suggest that a template-based structure elucidation is a helpful approach for elucidating the structures of peptides that belong to the same type. All structures were confirmed by MS.

Many lipo-tridecapeptides have shown an antimicrobial activity against pathogenic bacteria, including antibiotic-resistant *S. aureus* and *Enterococcus* spp. (Barsby et al., 2006; Yang et al., 2016; Wu et al., 2019). However, the high hemolytic activity of lipo-tridecapeptides has limited their potential for being developed as therapeutics (Figure 3; Li et al., 2018). In this study, one of the discovered novel lipo-tridecapeptides, brevibacillin 2V, showed no hemolytic activity at a high concentration of 128 mg/L (Figure 3). Moreover, the hemolytic activity of brevibacillins shows a positive correlation with their calculated hydrophobicity, i.e., brevibacillin I > brevibacillin > brevibacillin V > brevibacillin 2V (Figure 1C and Supplementary Figure 1). These results demonstrate that brevibacillin 2V shows a high potential to be further studied and developed as an alternative antibiotic for controlling antibiotic-resistant bacterial pathogens. In addition, the positive correlation of hemolytic activity and hydrophobicity provides a guideline for the synthesis of lipo-tridecapeptide analogs in future studies. The use of peptides as adjuvants for marketed antibiotics has attracted the attention of several researchers. Cochrane and Vederas (2014) reported that unacylated tridecaptin A1 acts as an effective sensitizer of Gram-negative bacteria to other antibiotics. More recently, Li et al. (2021) reported that outer-membrane-acting peptides showed a synergistic activity with lipid II-targeting antibiotics against Gram-negative pathogens. In the present study, brevibacillins showed a synergistic activity with marketed antibiotics against Gram-negative pathogens. These results suggest that more antibiotics can be tested for developing brevibacillins (at least, brevibacillin 2V) as adjuvants for other antibiotics to treat Gram-negative bacterial pathogen infections.

## Conclusion

In conclusion, in this study, we characterized a novel lipo-tridecapeptide, i.e., brevibacillin 2V, which was identified from *B. laterosporus* DSM 25 by genome mining and subsequently isolated and purified. Brevibacillin 2V has a strong antimicrobial activity against antibiotic-resistant Gram-positive bacterial pathogens, and it shows an effective synergistic activity with other antibiotics against Gram-negative bacterial pathogens. Notably, brevibacillin 2V has a much lower hemolytic activity and cytotoxicity towards eukaryotic cells than previously reported NRPs of the lipo-tridecapeptide family, including all known brevibacillins, which makes it a promising candidate for antibiotic development. In addition, brevibacillin 2V showed good stability in human plasma. This study provides a novel and promising antibiotic candidate (brevibacillin 2V) with low hemolytic activity, which can be used either directly as it is or serves as a template for further total synthesis and modifications.

## Data Availability Statement

The original contributions presented in the study are included in the article/Supplementary Material, further inquiries can be directed to the corresponding author/s.

## Author Contributions

OK and XZ conceived the project. XZ designed and carried out the experiments, analyzed the data, and wrote the manuscript. OK and EB supervised the work and corrected the manuscript. XW, RS, RK, and MW did the experimental work. All authors contributed to and commented on the manuscript text and approved its final version.

## Conflict of Interest

The authors declare that the research was conducted in the absence of any commercial or financial relationships that could be construed as a potential conflict of interest.

## Footnotes

1 https://www.sanquin.nl
