# Supplementary material for: Brevibacillin 2V, a Novel Antimicrobial Lipopeptide With an Exceptionally Low Hemolytic Activity
Source: Front Microbiol. 2021 Jun 17;12:693725. doi: 10.3389/fmicb.2021.693725 (PMC8245773; doi:10.3389/fmicb.2021.693725)
Supplement: Supplementary file 1 [file Data_Sheet_1.DOC]

Supplementary Material

Brevibacillin 2V, a novel antimicrobial lipopeptide with an exceptional low hemolytic activity

Xinghong Zhao ^1^, Xiaoqi Wang ^2^, Rhythm Shukla ^2, 3^, Raj Kumar ^3^, Markus Weingarth ^3^, Eefjan Breukink ^2^, Oscar P. Kuipers ^1^, *

^1^ Department of Molecular Genetics, Groningen Biomolecular Sciences and Biotechnology Institute, University of Groningen, Nijenborgh7, 9747 AG Groningen, The Netherlands.

^2^ Membrane Biochemistry and Biophysics, Bijvoet Centre for Biomolecular Research, Department of Chemistry, Faculty of Science, Utrecht University, Padualaan 8, Utrecht, The Netherlands.

^3^ NMR Spectroscopy, Bijvoet Centre for Biomolecular Research, Department of Chemistry, Faculty of Science, Utrecht University, Padualaan 8, Utrecht, The Netherlands.

* Correspondence: o.p.kuipers@rug.nl (Oscar P. Kuipers)


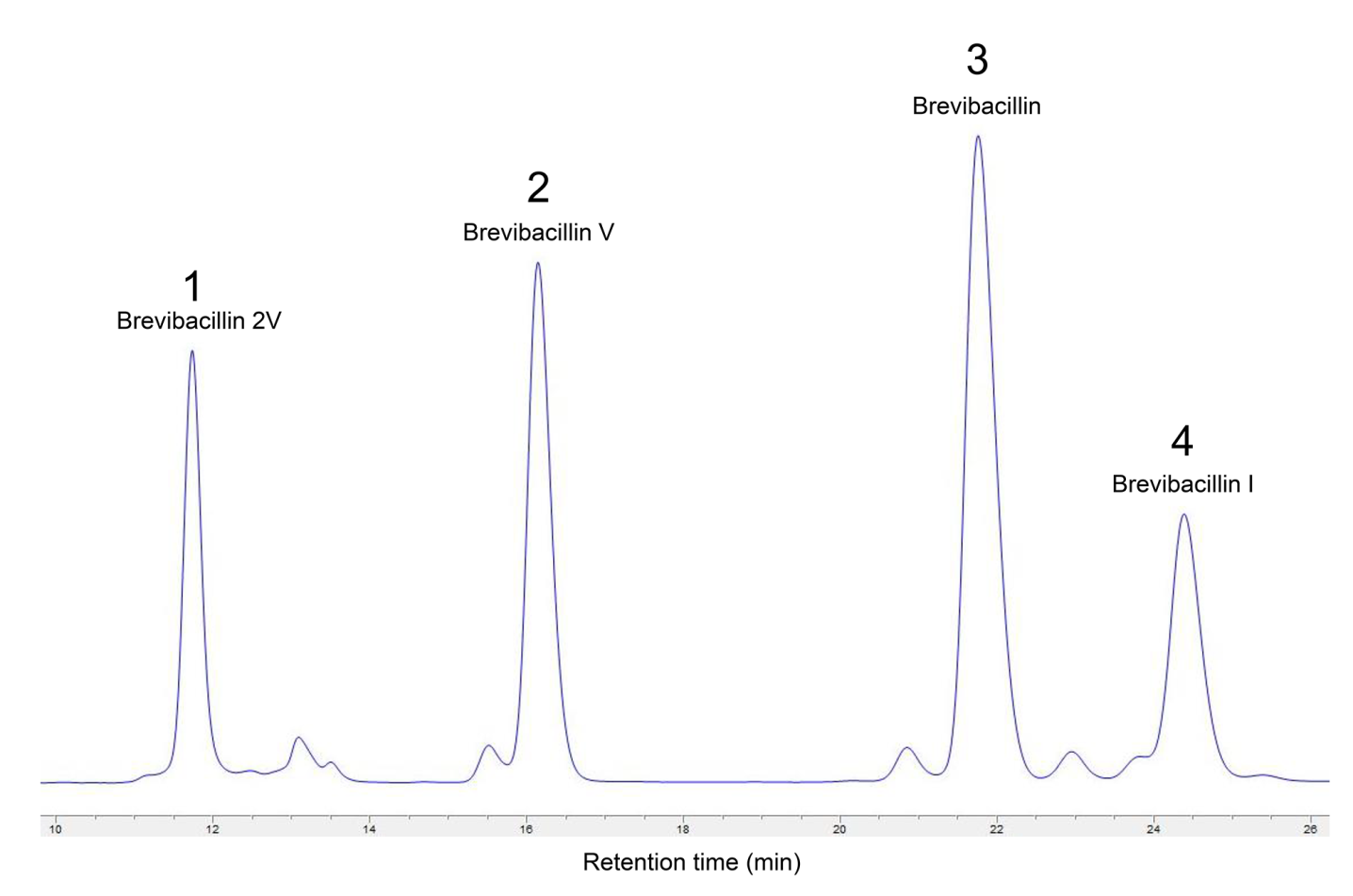


**Supplementary Figure 1.** The HPLC spectrum of brevibacillins. The longer retention time correlated with the higher hydrophobicity of brevibacillins, which shows hydrophobicity that brevibacillin I ＞ brevibacillin＞ brevibacillin V ＞ brevibacillin 2V. Compounds 1, 2, 3 and 4 were elucidated by further studies as brevibacillin 2V, brevibacillin V, brevibacillin and brevibacillin I, respectively.


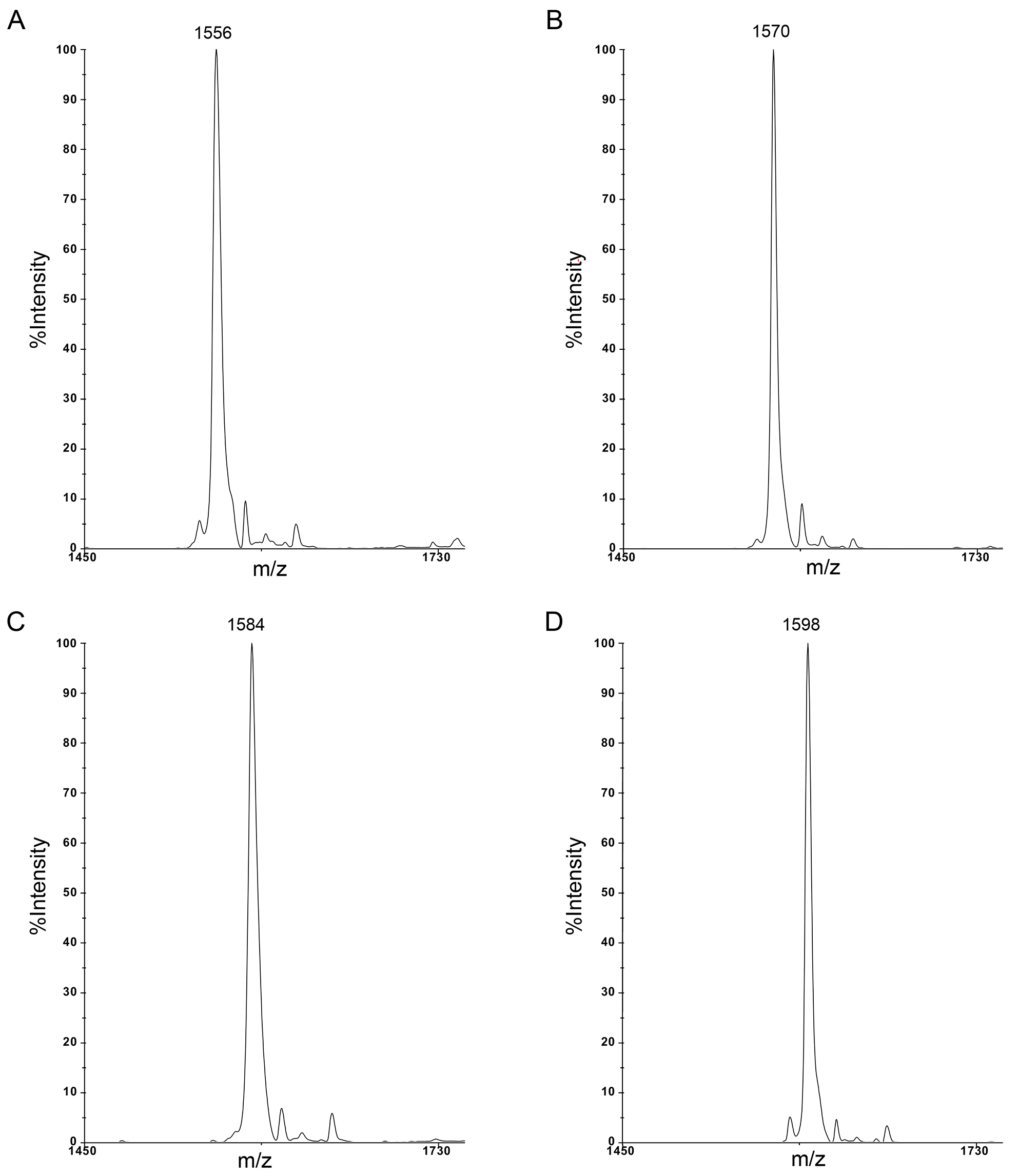


**Supplementary Figure 2.** MALDI-TOF spectrum of HPLC purified brevibacillins. **A**, brevibacillin 2V; **B**, brevibacillin V; **C**, brevibacillin; **D**, brevibacillin I.


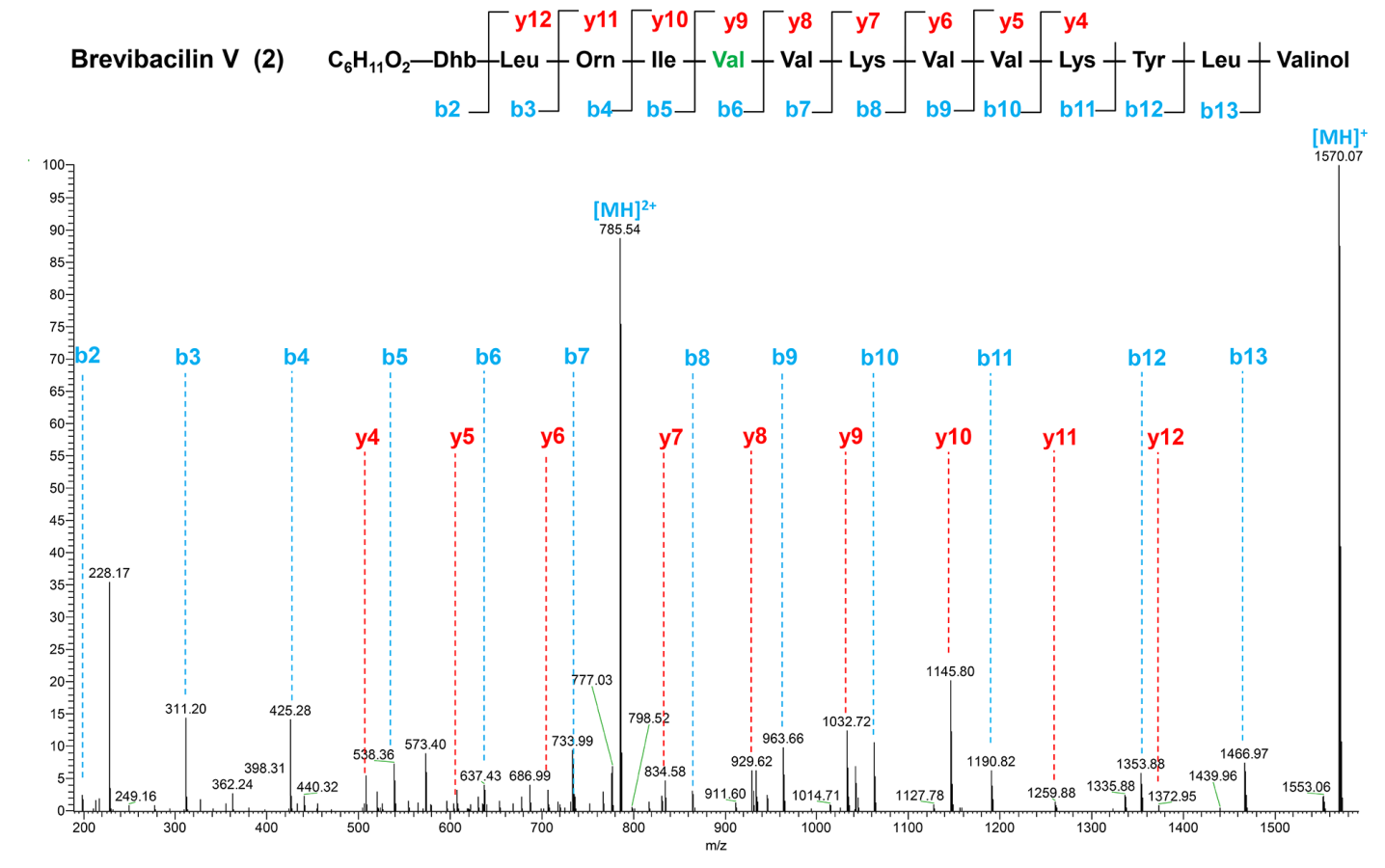


**Supplementary Figure 3.** LC-MS/MS spectrum and the proposed structure of brevibacillin V (2). Fragment ions are indicated.


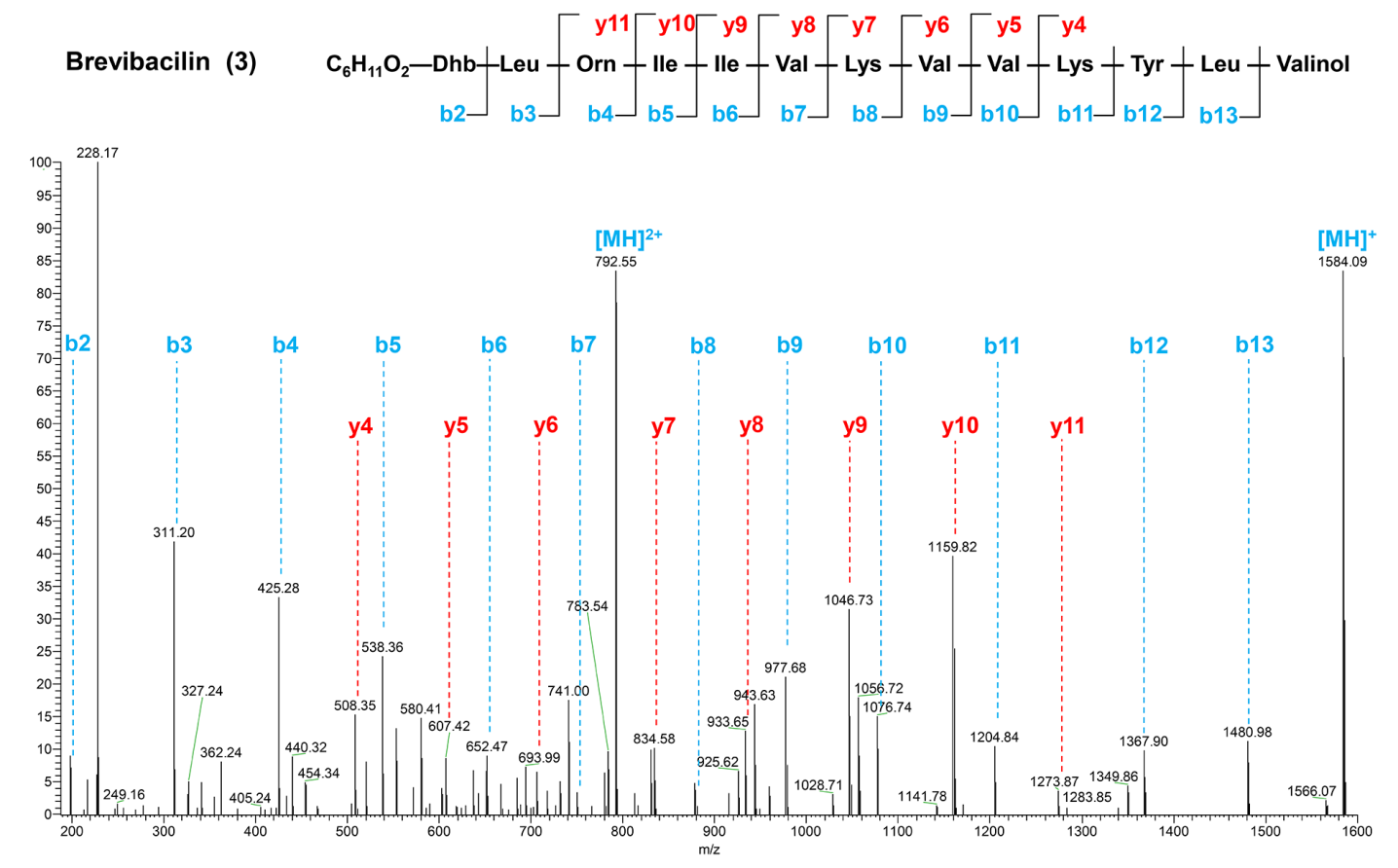


**Supplementary Figure 4.** LC-MS/MS spectrum and the proposed structure of brevibacillin (3). Fragment ions are indicated.


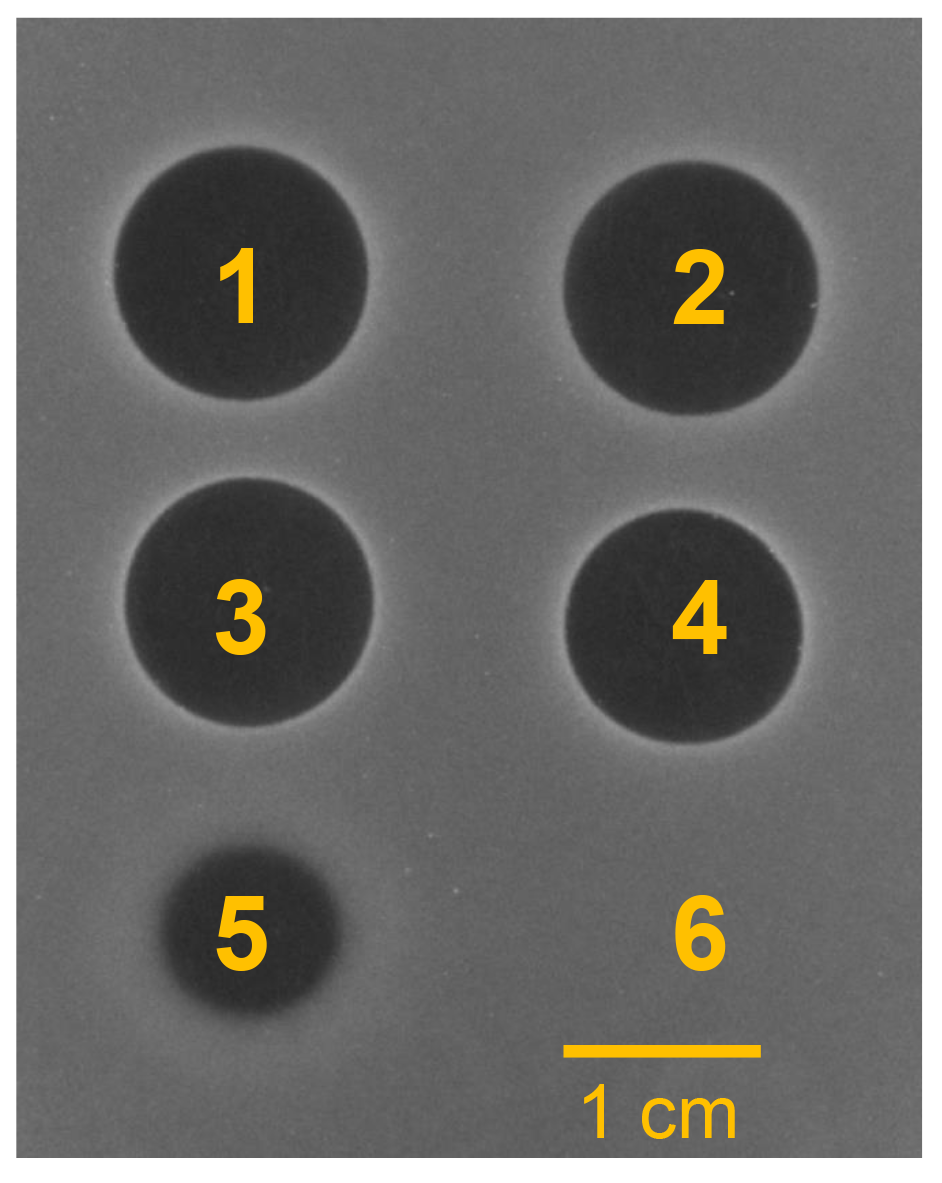


**Supplementary Figure 5.** Antimicrobial activity of brevibacillins against *Staphylococcus aureus* ATCC15975 (MRSA). Spot-on-lawn assay with *S. aureus* (MRSA). 1, brevibacillin; 2, brevibacillin V; 3, brevibacillin I; 4, brevibacillin 2V; 5, nisin; 6, Milli-Q water. Brevibacillins and nisin were added at a concentration of 100 μg/mL and with a volume of 6.8 μL.

**Supplementary Table 1** Strains used in this study.

| Strains | Characteristics and purpose | Source |
| --- | --- | --- |
| *Brevibacillus laterosporus* | DSM 25, host strain for production of brevibacillins | BCCM |
| *Bacillus cereus* | ATCC14579, indicator strain | ATCC |
| *Enterococcus faecalis* | LMG16216 (VRE), indicator strain | BCCM |
| *Staphylococcus aureus* | ATCC15975 (MRSA), indicator strain | ATCC |
| *Enterococcus faecium* | LMG16003 (VRE), indicator strain | BCCM |
| *Acinetobacter baumannii* | ATCC17978, indicator strain | ATCC |
| *Escherichia coli* | ATCC25922, indicator strain | ATCC |
| *Klebsiella pneumoniae* | LMG20218, indicator strain | BCCM |
| *Pseudomonas aeruginosa* | LMG6395, indicator strain | BCCM |

**Supplementary Table 2** Synergistic effect between brevibacillin V and antibiotics.

| Microoganism | Antibiotic | MIC(mg/L) at brevibacillin V concentrations of ^a^ | | | |  | FICI |
| --- | --- | --- | --- | --- | --- | --- | --- |
|  |  | 0 | 1 | 2 | 4 |  |  |
|  |  |  |  |  |  |  |  |
| *E.coli*  ATCC 25922 | Nalidixic acid | 2 | 2 | 1 | 0.5 |  | 0.375 |
|  | Rifampicin | 4 | 4 | 2 | 1 |  | 0.375 |
|  | Amikacin | 4 | 1 | 1 | 0.5 |  | 0.250 |
|  | Azithromycin | 2 | 2 | 1 | 1 |  | 0.563 |
|  |  |  |  |  |  |  |  |
| *A. baumannii* ATCC17978 | Nalidixic acid | 32 | 32 | 32 | 32 |  | 1.016 |
|  | Rifampicin | 32 | 16 | 16 | 16 |  | 0.516 |
|  | Amikacin | 16 | 4 | 2 | **0.5** |  | **0.094** |
|  | Azithromycin | 16 | 16 | 16 | 16 |  | 1.016 |
|  |  |  |  |  |  |  |  |
| *P. aeruginosa* LMG6395 | Nalidixic acid | 256 | 128 | 128 | 128 |  | 0.516 |
|  | Rifampicin | 32 | 16 | 16 | 16 |  | 0.516 |
|  | Amikacin | 2 | 0.5 | 0.5 | 0.5 |  | 0.266 |
|  | Azithromycin | 128 | 64 | 64 | 64 |  | 0.516 |
|  |  |  |  |  |  |  |  |
| *K. pneumoniae* LMG20218 | Nalidixic acid | 32 | 32 | 32 | 32 |  | 1.016 |
|  | Rifampicin | 64 | 16 | 16 | 16 |  | 0.266 |
|  | Amikacin | 0.5 | 0.5 | 0.5 | 0.5 |  | 1.016 |
|  | Azithromycin | 16 | 16 | 16 | 16 |  | 1.016 |

^a^The MIC was determined by broth microdilution. FICI, fractional inhibitory concentration index [FICI = (MIC compound A in combination with B / MIC compound A) + (MIC compound B in combination with A / MIC compound B) ]. The FICI value suggests a synergistic (≤ 0.5), addictive (> 0.5 - 1), no interaction (1 - 4), and antagonism (> 4) effect of the two compounds. The bold font indicates the best synergy effect among the tests.

**Supplementary Table 3** Synergistic effect between brevibacillin and antibiotics.

| Microoganism | Antibiotic | MIC (mg/L) at brevibacillin concentrations of ^a^ | | | |  | FICI |
| --- | --- | --- | --- | --- | --- | --- | --- |
|  |  | 0 | 1 | 2 | 4 |  |  |
|  |  |  |  |  |  |  |  |
| *E.coli*  ATCC 25922 | Nalidixic acid | 2 | 1 | 1 | 0.5 |  | 0.375 |
|  | Rifampicin | 4 | 2 | 1 | 0.5 |  | 0.250 |
|  | Amikacin | 4 | 0.5 | 0.5 | 0.5 |  | 0.156 |
|  | Azithromycin | 2 | 1 | 0.5 | 0.125 |  | 0.188 |
|  |  |  |  |  |  |  |  |
| *A. baumannii* ATCC17978 | Nalidixic acid | 32 | 32 | 32 | 16 |  | 0.625 |
|  | Rifampicin | 32 | 8 | 8 | 8 |  | 0.281 |
|  | Amikacin | 16 | 2 | 0.5 | **0.25** |  | **0.094** |
|  | Azithromycin | 16 | 8 | 8 | 8 |  | 0.531 |
|  |  |  |  |  |  |  |  |
| *P. aeruginosa* LMG6395 | Nalidixic acid | 256 | 128 | 128 | 128 |  | 0.516 |
|  | Rifampicin | 32 | 16 | 8 | 8 |  | 0.281 |
|  | Amikacin | 2 | 0.5 | 0.5 | 0.5 |  | 0.266 |
|  | Azithromycin | 128 | 64 | 64 | 64 |  | 0.516 |
|  |  |  |  |  |  |  |  |
| *K. pneumoniae* LMG20218 | Nalidixic acid | 32 | 32 | 16 | 16 |  | 0.563 |
|  | Rifampicin | 64 | 16 | 16 | 16 |  | 0.281 |
|  | Amikacin | 0.5 | 0.5 | 0.5 | 0.5 |  | 1.031 |
|  | Azithromycin | 16 | 16 | 16 | 16 |  | 1.031 |

^a^The MIC was determined by broth microdilution. FICI, fractional inhibitory concentration index [FICI = (MIC compound A in combination with B / MIC compound A) + (MIC compound B in combination with A / MIC compound B) ]. The FICI value suggests a synergistic (≤ 0.5), addictive (> 0.5 - 1), no interaction (1 - 4), and antagonism (> 4) effect of the two compounds. The bold font indicates the best synergy effect among the tests.

**Supplementary Table 4** Synergistic effect between brevibacillin I and antibiotics.

| Microoganism | Antibiotic | MIC (mg/L) at brevibacillin I concentrations of ^a^ | | | |  | FICI |
| --- | --- | --- | --- | --- | --- | --- | --- |
|  |  | 0 | 1 | 2 | 4 |  |  |
|  |  |  |  |  |  |  |  |
| *E.coli*  ATCC 25922 | Nalidixic acid | 2 | 1 | 0.5 | 0.5 |  | 0.313 |
|  | Rifampicin | 4 | 2 | 2 | 2 |  | 0.531 |
|  | Amikacin | 4 | 1 | 1 | 1 |  | 0.281 |
|  | Azithromycin | 2 | 1 | 1 | 0.5 |  | 0.375 |
|  |  |  |  |  |  |  |  |
| *A. baumannii* ATCC17978 | Nalidixic acid | 32 | 16 | 16 | 16 |  | 0.516 |
|  | Rifampicin | 32 | 16 | 16 | 16 |  | 0.516 |
|  | Amikacin | 16 | 8 | 4 | **0.25** |  | **0.078** |
|  | Azithromycin | 16 | 8 | 8 | 8 |  | 0.516 |
|  |  |  |  |  |  |  |  |
| *P. aeruginosa* LMG6395 | Nalidixic acid | 256 | 64 | 64 | 64 |  | 0.266 |
|  | Rifampicin | 32 | 16 | 16 | 16 |  | 0.516 |
|  | Amikacin | 2 | 0.5 | 0.5 | 0.5 |  | 0.266 |
|  | Azithromycin | 128 | 32 | 32 | 32 |  | 0.266 |
|  |  |  |  |  |  |  |  |
| *K. pneumoniae* LMG20218 | Nalidixic acid | 32 | 16 | 16 | 16 |  | 0.516 |
|  | Rifampicin | 64 | 16 | 16 | 16 |  | 0.266 |
|  | Amikacin | 0.5 | 0.5 | 0.5 | 0.25 |  | 0.563 |
|  | Azithromycin | 16 | 16 | 16 | 16 |  | 1.016 |

^a^The MIC was determined by broth microdilution. FICI, fractional inhibitory concentration index [FICI = (MIC compound A in combination with B / MIC compound A) + (MIC compound B in combination with A / MIC compound B) ]. The FICI value suggests a synergistic (≤ 0.5), addictive (> 0.5 - 1), no interaction (1 - 4), and antagonism (> 4) effect of the two compounds. The bold font indicates the best synergy effect among the tests.
